# Determinants of Microbial Contamination of Street-Vended Chicken Products Sold in Nairobi County, Kenya

**DOI:** 10.1155/2020/2746492

**Published:** 2020-02-14

**Authors:** Beatrice J. Birgen, Lucy G. Njue, Dasel M. Kaindi, Fredrick O. Ogutu, Joshua O. Owade

**Affiliations:** ^1^Department of Food Science, Nutrition and Technology, University of Nairobi, P.O. Box 29053-00625 Kangemi, Nairobi, Kenya; ^2^Food Technology Division, Kenya Industrial Research and Development Institute, P.O. Box 30650, GPO, Nairobi, Kenya

## Abstract

Food safety problems pose a great threat to the health of consumers with the greatest burden in developing countries. Street-vended foods play a key role in providing many urban dwellers with cheap, nutritious, and accessible food, but when prepared in an unhygienic and unregulated environment, they could contribute to increased food safety burden. The study investigated the microbiological recovery of work surfaces and chicken sold in Korogocho and Kariobangi North slums in Nairobi County as well as evaluating vendors' hygiene and food safety practices. This is a cross-sectional study on an exhaustive sample size of 15 vendors, and swabs of the equipment and work surfaces and chicken were taken for microbial analysis. An exhaustive sample size of 15 vendors was selected for the study. The results showed that most vendors operate under unhygienic conditions. Microbial results revealed that raw portions of chicken had the highest contamination with all the four tested microorganisms (*p* < 0.05). The level of *E. coli* ranged from 6.42 ± 1.64 to 2.22 ± 1.88; *Salmonella* spp., 6.42 ± 1.64 to 2.22 ± 1.88; *Staphylococcus aureus*, 6.92 ± 1.32 to 2.86 ± 1.61; and *Campylobacter jejuni*, 8.95 ± 0.94 to 4.66 ± 2.67 log CFU/g in raw and cooked chicken samples, respectively. The predictors of *E. coli* contamination were the presence of pests and flies, unclean vending place, vending environment littered with waste, washing of hands by the vendor, and lack of appropriate clothing among the vendors at *R*^2^ of 0.33. The vendor practices and environmental hygiene of the vending place would not significantly (*p* > 0.05) predict contamination with *Campylobacter* and *Staphylococcus*. Consequently, there is a need to regulate the informal food processing and marketing channels, besides trainings, infrastructural development, and code of practice and inspections which are recommended in order to enhance the quality and safety standards of street-vended chicken products.

## 1. Introduction

The availability and comparatively low prices of street-vended foods relative to the processed and already-packaged foods have increased their reliability to customers [1]. This has also increased their popularity and preference by low- and medium-income earners in developing countries [2]. The appreciation of street-vended foods can be attributed to their convenience and distinctive flavors in addition to maintenance of the population's nutritional status. In most developing countries where unemployment rates are high, low-income urban dwellers are assured of their food security by the availability of street-vended foods which also act as a form of generating income and employment [3–5].

In spite of numerous advantages provided by street-vended foods, they have been reported to present serious safety and health concerns to consumers and food handlers. This is due to their diversity, inadequate food safety knowledge and practices, insufficient basic hygiene, and lack of public awareness [6, 7]. Insufficient processing and storage facilities, inadequate basic infrastructure, difficulties in regulating huge numbers of vendor operations, and nonpermanent nature of street vending activities aggravate the public health risks posed by street-vended foods [8]. The major possible health hazards related to foods sold in the streets include environmental contamination, use of unauthorized chemical additives, parasite transmissions, pesticide residues, and microbial contamination [9]. Although pH, temperature, type of food, preparation methods, handling, holding time, and availability of clean water influence the food risks, the storage life of street-vended foods is determined by the interaction of physical, microbial, and chemical factors [10].

The common foodborne pathogens associated with street-vended foods include *Clostridium perfringens*, *Escherichia coli*, *Shigella* spp., *Campylobacter jejuni*, *Staphylococcus aureus*, *Salmonella* spp., and *Bacillus cereus* [11]. The preparation techniques of street-vended foods include chicken frying, baking, boiling, fermenting, braising, roasting, or juicing [12]. Vendors often serve the foods without further reheating; thus, the aforementioned microorganisms are prevalent in these foods. Additionally, the improvisation of preparation techniques including display of products exacerbates the food safety risks posed by the microorganisms [13]. The prevalent foodborne diseases are also a result of limited training and poor food safety and handling knowledge among the vendors [2, 14].

Several studies about street-vended foods in Kenya and other developing countries revealed the presence of pathogens as well as favorable conditions to allow their proliferation [15–17]. However, the information on the microbiological safety and quality of street-vended chicken products in Nairobi City is very scanty. The objective of this study was therefore to evaluate the microbial safety of street-vended chicken products that are sold in Nairobi City, Kenya.

## 2. Material and Methods

### 2.1. Study Area, Design, and Sampling Procedure

**Figure 1:**
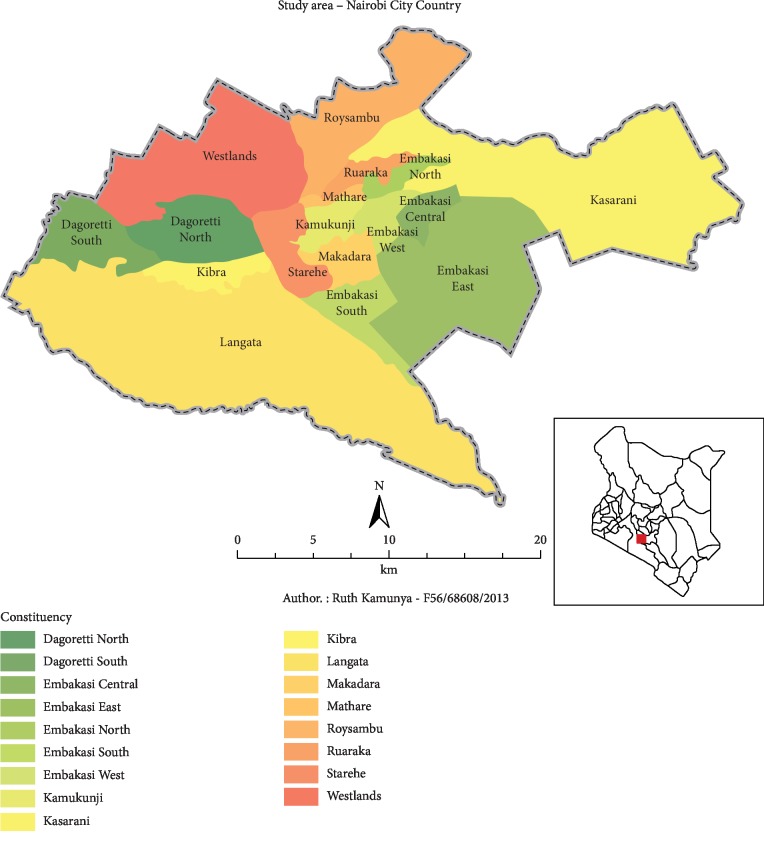
Map of Nairobi County [19].

A cross-sectional study was conducted in informal settlements: Korogocho (1.2504°S and 36.8909°E GPS coordinates) and Kariobangi North (1.2534°S and 36.88815°E GPS coordinates), Nairobi County (1.2921°S and 36.8219°E GPS coordinates, Figure 1) of Kenya. The 2009 national population census estimated the population of Nairobi County to be over three million, with over half of the population living in slums [18]. Street vendors of chicken from Korogocho and Kariobangi North were sampled in the survey.

### 2.2. Data Collection Tools and Procedure

A food safety checklist and direct observation were employed as data collection tools. Content of the questionnaire included issues addressing sociodemographic characteristics, health status and personnel hygiene, food handling practices and food safety knowledge of the vendors, and access to hygienic water supply and other sanitary facilities.

### 2.3. Sampling and Sample Collection

Nairobi County was purposively selected for the study because of its populous nature. Korogocho and Kariobangi North areas were also purposively selected for the study as they have largely informal settlements with the population being those of low income. A total of fifteen vendors were exhaustively sampled and included in the study with the food safety and hygiene practices evaluated using a food safety checklist. The snowballing sampling technique was used to locate all the vendors. Swabs were collected from the hands of the personnel and equipment (knives, storage container, and display surfaces) and raw and cooked chicken meat products subjected to microbial analysis. The cooked samples were defined as the portion of chicken that had been subjected to deep frying which was the technique in use. Raw chicken were defined as uncooked chicken portions.

The swabbing technique employed procedures described by SOP [20] and Bersisa et al. [21]. Swabs were soaked in peptone water and rubbed on a surface of 5 cm by 10 cm first horizontally and then vertically. The swabs were stored in 9 ml diluent. All swab and chicken meat samples were collected in sterile polythene bags followed by transportation to the laboratory of the Department of Food Science and Technology of the University of Nairobi. The samples were stored at a temperature of 4°C and analyzed within 2 hours of collection.

### 2.4. Checklist for Food Handling Practices

Food safety practices of all the vendors in the streets were assessed using a checklist that was adapted from checklists used by previous researchers [22, 23]. The demographic data included location, sex, educational level, age, and occupation. The hygienic practices were evaluated using “yes” or “no” which were then expressed in proportions. The selection of participants for this part of the study was based on the same methodology as for the selection of vendors for the food safety and attitude questionnaire. The aim of the study was clearly elaborated to respondents; after which, the volunteers signed the consent form and filled in the questionnaire.

## 3. Microbial Analysis

### 3.1. Determination of *Escherichia coli*

Based on the ISO method 9308-1:2000 [24], the *E. coli* was accordingly enumerated. About 10 g of the chicken sample was homogenized in 90 ml peptone water. In the case of swab samples, 10 ml sample was used. Decimal serial dilutions of the homogenized solution in sterile peptone water were prepared and plated in duplicate on the Chromogenic Coliform Agar media. Blue-green colonies for *E. coli* were counted after 48 hours of incubation at 44°C. The number of colony-forming units (CFU) determined using the colony count technique of presumptive *E. coli* per gram of sample was calculated.

### 3.2. Determination of *Salmonella*

The ISO method ISO 6579 [25, 26] was used to enumerate the *Salmonella* species. Raw and cooked meat samples of 10 g were each weighed, homogenized in 90 ml buffered peptone water, and incubated at 37 ± 1°C for 18 ± 2 hours, whereas for the swab samples from knives, display surfaces, hand, and storage containers, 10 ml of the soaked swab and diluent had been used. From preenrichment broth, the inoculums were transferred to Rappaport-Vassiliadis broth and selenite cysteine broth and then incubated at 41.5 ± 1°C and 37 ± 1°C for 24 hours for selective enrichment. A loopful of the selective enrichment was streaked onto solid selective media: xylose lysine deoxycholate agar (XLD). XLD agar was incubated at 37 ± 1°C and observed after 24 ± 3 hours for typical *Salmonella* transparent red halo and a black centre. The colony count technique was used in the enumeration of the microbial population.

### 3.3. Determination of *Staphylococcus aureus*

The EN ISO method ISO 6888-1:1998 [27] was used for the detection and enumeration of *Staphylococcus aureus.* About 10 g of raw and cooked meat samples homogenized in 90 ml peptone water and 10 ml of the soaked swab sample were used to prepare serial dilutions. In a sterile pipette, 0.1 ml of the appropriate sample test dilutions was transferred in duplicate onto the Baird Parker agar (BPA). The plates were then incubated at 35-37°C for 24 ± 2 hours and then reincubated for further 24 ± 2 hours. Observation ensued for typical colonies appearing black or grey, shining and convex, and 1-1.5 mm in diameter after 24 hours and 1.5-2.5 mm after 48 hours of incubation, surrounded by a clear zone but partially opaque zone. The colony count technique was used to enumerate the microbes. The coagulase-positive staphylococci were then expressed as CFU/g of the sample.

### 3.4. Determination of *Campylobacter jejuni*

Analysis was conducted according to ISO 10272-1:2017 [28] procedures which specify a horizontal method for the detection and enumeration of *Campylobacter* spp.

### 3.5. Data Analysis

Both the microbial count and survey data were analyzed in IBM SPSS version 21. The frequencies and descriptive statistics such as mean, standard deviation, and minimum and maximum for the sociodemographic score and score for food practices were obtained. The linear regression test was used to test the predictability of food safety knowledge by the sociodemographic factors. The microbial counts were transformed into log CFU. The ANOVA test was used to test for statistical difference in the microbial counts with statistically different means separated using Tukey's Honestly Significant Difference (HSD) test. Statistical significance was tested at *p* < 0.05.

## 4. Results and Discussions

The hygiene and food handling practices that determined the microbial safety of chicken products vended in Nairobi City are shown in Tables 1 and 2. In the study, only 33% of the vending places were sheltered while 60.0% of them were not clean. In other studies conducted by Chukuezi [29], it was reported that only 29% of the vending sites had a canopy which is consistent with this study. These conditions allow the dust and exhaust fumes to find their way into most products causing contamination. Holding foods at ambient temperature beyond 4-6 hours poses a great risk to public health as these conditions can contribute to high microbial counts of the foods [30]. According to Muinde and Kuria [22], these kinds of structures do not adequately protect street foods from vehicles' smoke and surrounding dust which carry many pathogenic microbes, hence posing a health hazard to consumers of such foods. In contrast to the present findings where 87% of vendors prepared their chicken products on site, only 10% of vendors in South Africa were preparing on the vending sites [31]. This preference can be explained by the lack of refrigeration and storage facility for their cooked chicken before sale. Lack of clean clothing (60%), lack of appropriate clothing for food preparation (47%), and long nails with visible dirt of some vendors increased chances of cross contamination and posed a health hazard to consumers of street-vended chicken products. Personal hygiene is crucial while handling food because human beings have been reported to be the major contamination sources of foods [9, 29]. Proper personal cleanliness, hygiene, and safe handling practices of food should be maintained by all food handlers to avoid microbial contamination of food. The hands should be kept clean, fingernails should be cut short, working garments should be kept clean, and the hair should be covered with nets or cover to ensure safety of the prepared food [32].

Six in every ten (60%) of the studied vending places were not clean, and 33% had pests and flies in the surroundings. Eight in every ten (80%) and two-thirds (67%) lacked hand washing and waste disposal facilities, respectively. These poor handling practices can be attributed to lack of potable water and washing facilities on the sites and lack of awareness about food handling and safety [9]. These findings corroborate the findings of Badrie et al. [33] and Muyanja et al. [6] in Trinidad and Uganda, respectively. Lack of waste disposal facilities prompts the vendors to discard their wastes behind the vending sites and in the streets which attract flies and pests that act as vectors for pathogenic microorganisms [23]. The current survey showed that the health risk of consuming street-vended chicken products is high due to the unhygienic practices which affected the overall safety. However, the study by Von Holy and Makhoane [34] found out that vendors of street foods have the potential to prepare comparatively hygienic and safe products with lower microbial counts to curb the spread of foodborne diseases. The observations in this study also differ from those in the survey conducted in the Philippines by Azanza et al. [35] that reported proper hand washing and food handling practices due to availability of adequate hand washing facilities within the vending sites and relatively higher knowledge levels among the vendors. In the present study, the vending sites lacked available hand washing and sanitary facilities as the vending sites were along the roads.

The survey also revealed that none of the interviewed street vendors handled food with any special equipment or disposable gloves and 20% did not wash their hands during handling and preparation of chicken products. It was also observed that all the vendors served chicken products while at the same time handling money. These findings are in agreement with the observations made by Muinde and Kuria [22] in Kenya and Omemu and Aderoju [36] in Nigeria who noted that street vendors handled money as well as food with bare hands. Hands are potential vectors for transmitting pathogenic microbes such as *Staphylococcus aureus*; hence, the above practices are major concerns to the safety of consumers of street-vended foods [37]. It is recommended that food handlers should not handle food with bare hands and handle money simultaneously to avoid incidences of cross contamination that can be health risk [38]. Moreover, FAO et al. [39] and WHO/FAO (1999) recommend the use of disposable gloves and clean tongs, forks, or spoons during handling of food. Six in every ten (60.0%) of the vendors also did not cover their utensils while only 20.0% covered chicken products awaiting sale (Table 2). This is in line with the findings of Samapundo et al. [23] who observed that street vendors in Haiti were not covering their foods hence exposing them to dust containing microbes which contaminated the foods.

Most vendors used dirty water to clean the utensils which was normally recycled and reused severally hence increasing chances of cross contamination and subsequent transfer of pathogens to the products. Reused water contains diffused organic materials which act as a culture medium that allows proliferation of several pathogenic microorganisms hence compromising food safety. These findings are in accordance with the observations of Chukuezi [29] who reported that all street vendors in Owerri, a region in Nigeria, handled money and foods concurrently and 48% washed utensils with dirty water and handled foods with bare hands which compromised the food microbial safety. Based on the findings by Gadaga et al. [40], knowledge on food safety issues raises adherence to public health regulations. The preparation of street foods under the aforementioned unsanitary and unhygienic conditions poses great health risks to consumers as the conditions predispose them to outbreaks of foodborne diseases [36].

The microbial results are summarized in Table 3. Raw portions of chicken products had the highest contamination with all the four tested microorganisms (*p* < 0.05). The presence of such high microbial counts can be attributed to improper handling of raw chicken products and inadequate storage conditions [41]. Regression analysis showed that the presence of pests, unclean vending place, littered vending, appropriate clothing, clean clothing, and covering of utensils' environment with beta values of 2.6, 4.2, 1.9, 2.2, 2.2, and 2.4, respectively, were positive predictors of *Salmonella* contamination, accounting for 22% variation in the dependent variable (*p* < 0.005, *R*^2^ = 0.22).

*y* = 7.3 + 2.6*a* + 4.2*b* + 1.9*c* + 2.2*c* + 2.2*d* + 2.4*e*, where *a*, *b*, *c*, *d*, and *e* represent the variables presence of pests, unclean vending place, littered vending, appropriate clothing, clean clothing, and covering of utensils' environment, respectively.

The predictors of *E. coli* contamination were the presence of pests and flies, unclean vending place, vending environment littered with waste, washing of hands by the vendor, and lack of appropriate clothing among the vendors at *R*^2^ of 0.33.

*y* = 13.5 + 2.4*a* + 3.8*b* + 2.9*c* − 1.6*c* + 1.6*d*, where *a*, *b*, *c*, and *d* represent the variables presence of pests and flies, unclean vending place, vending environment littered with waste, washing of hands by the vendor, and lack of appropriate clothing, respectively. The vendor practices and environmental hygiene of the vending place would not significantly (*p* > 0.05) predict contamination with *Campylobacter* and *Staphylococcus*.

The level of *E. coli* ranged from 6.42 ± 1.64 log_10_ CFU/g in raw chicken to 2.22 ± 1.88 log_10_ CFU/g in cooked chicken products, higher than the regulatory level of 1 log_10_ CFU/g set by the Kenya Bureau of Standards (KEBS) in Kenya [13]. On the other investigated samples, hands, knives, surfaces, and storage containers, the microbial counts were highest on the operating surfaces (3.68 ± 1.82 log_10_ CFU/g) showing that most of the chicken products were a potential risk to public health. However, there was no significant (*p* < 0.05) difference in the microbial counts of cooked samples, knives, surfaces, containers, and hand. The high microbial counts relative to the regulatory level (10 CFU/g) in these samples are attributable to temperature and time abuse during street vending and cross contamination as a result of poor handling and improper vending practices as well as lack of cold storage facilities during sale [9]. Since these microorganisms are indicators in the assessment of food safety, high counts show the likely presence of pathogenic microbes due to unhygienic handling of food [41]. Comparable findings were reported by Haranisho et al. (2005) and Mohammed (2017) who reported high counts of *E. coli* in street-vended food products indicating inappropriate holding temperatures. The results are also in line with the findings of Gitahi et al. [13] who observed 3.45 log_10_ CFU/g in raw meat sold along the streets of Nairobi, Kenya. *E.coli* usually proliferates in the humans' GIT and is also found in faeces; hence, its presence in food also indicates fecal contamination either from materials used or at some point during preparations [42]. On the contrary, Mafune et al. [1] reported absence of *E. coli* in the sampled street-vended foods and attributed this to adequate processing, good quality of water used, and personal hygiene and storage temperatures.

*Salmonella* spp. were detected in cooked chicken products and on hands, knives, working surfaces, and storage containers (Table 3). However, the difference in counts of *Salmonella* spp. was not statistically significant (*p* > 0.05). The level detected in cooked chicken (2.22 ± 1.88 log_10_ CFU/g) is higher than the regulatory level of ready-to-eat food in Kenya whereby it should be undetectable [43]. The presence of *Salmonella* in these chicken products and working areas can be considered potentially hazardous to vendors and customers, and hence, these products are not acceptable for consumption [44]. Their presence is attributable to poor hygiene by vendors and unsanitary facilities on the vending site [45]. Comparable results were observed by Tesfaye et al. [10] when they examined the microbial safety of street-vended foods in Jigjiga City of Ethiopia. Similar results were also reported by Tambekar et al. [46] in India and Tassew et al. [17] in Ethiopia when street-vended foods were examined. This implies that notwithstanding the type of street-vended food, concerns of safety still remain. The current findings disagree with the observations of Kariuki [15] who did not detect *Salmonella* in foods vended in the streets of Gikomba and Githurai in Nairobi, Kenya. The difference can be explained by the informal setting that is usually known for unhygienic operating environment [47]. Other studies reported no *Salmonella* in the entire street-vended ready-to-eat food samples that were analyzed in Qatar [47, 48].

*Staphylococcus aureus* was found to be the highest on the hands of street food vendors (4.85 ± 1.00 log_10_ CFU/g). The cooked samples had a staphylococcal contamination level of 2.86 ± 1.61 log_10_ CFU/g which is within the acceptable national regulatory levels in Kenya [13]. The presence of *Staphylococcus* counts in chicken products and the vending site indicated the presence of poor hygienic and food handling practices as well as cross contamination, which is associated with discharges from clothing and human beings, human skin, dirty hands, mouth, nose, and utensils [10]. These results are in line with the observations of Badrie et al. [33] who reported 4.2 log CFU/g of *Staphylococcus aureus* counts in meat on a survey conducted in Trinidad and Tobago. On the contrary, Mafune et al. [1] and Ng et al. [49] reported absence of *Staphylococcus* counts in ready-to-eat street-vended foods in Thohoyandou, South Africa, and Hong Kong, respectively. The absence of staphylococcal counts denotes hygienic and proper handling practices of foods [13].

*Campylobacter jejuni* counts were the highest on the food display surfaces (6.84 ± 0.71 log_10_ CFU/g). The counts on the storage container, working surfaces, and hands did not differ significantly (*p* < 0.05). The counts on the knives and cooked chicken were also not statistically significant (*p* < 0.05). This can be attributed to cross contamination especially through hands and unsanitary conditions on the vending sites. According to FAO et al. [39], hands are the most utized means of transmission of microorganism from skin, noses, and faeces to the ready-to-eat foods. Epidemiological studies about *Campylobacter jejuni* have revealed that they are capable of surviving on surfaces and finger tips for differing periods and at times even after washing hands [9]. Hands should therefore be washed thoroughly before the work is started, instantly after visiting washrooms or after handling any materials with the potential of transmitting diseases [50]. In this study, however, washing of hands during and after the aforementioned activities was not practiced and the vending sites lacked portable water and washing facilities which can account for the high number of *Campylobacter jejuni* in the chicken products. According to Tesfaye et al. [10], *Campylobacter jejuni* can be isolated from vendors of street foods with poor sanitary practices and control and they can transfer these pathogenic and hazardous microbes to foods. The present findings are also in agreement with the observations of Cardinale et al. [51] and Haileselassie et al. [52] who reported that *Campylobacter* contaminated poultry products sold on the streets of Darkar City in Senegal, and they attributed this to unhygienic and unsanitary conditions on the vending sites. Poultry including chicken is also a known source of *Campylobacter* species, and the handling in slaughter houses is also a source of contamination of the meat [52, 53]. With improper handling of cooked and uncooked chicken, cross contamination occurs.

## 5. Conclusion

These findings demonstrate that chicken products sold in the streets of Nairobi constitute a potential health hazard to consumers because of high pathogenic bacterial counts such as *E. coli*, *Salmonella* spp., and *C. jejuni* isolated from the products that rendered them microbiologically unsafe and unacceptable. Their presence in ready-to-eat foods indicates a great risk to handlers and consumers and degrades quality of the food rendering it unfit and unsafe for consumption by humans. The county government and the public health department should also create awareness among street vendors and consumers through regular trainings on food safety and hygienic practices in food handling. The government needs to provide basic infrastructures and establish proper code of practice to improve the working conditions of street vendors.

## Figures and Tables

**Table 1:** Hygiene practices of the street vendors of the chicken products (*n* = 15).

Hygiene practices	Yes (percentage)
Vendor having appropriate clothing for food preparation	8 (53)
Vendor having clean clothing	6 (40)
Vendors nails kept short	14 (93)
Vendor handling food with bare hands	15 (100)
Sheltered vending place	5 (33)
Preparation of food on site	13 (87)
Evidence of pests and flies	5 (33)
Clean vending place	6 (40)
Presence of hand washing facilities	3 (20)
Availability of waste disposal facilities	5 (33)
Availability of closed bins for waste disposal	3 (20)

**Table 2:** Food handling practices of street vendors of chicken products (*n* = 15).

Vendor practices	Yes (%)
Vendor washes hands during food handling and preparation	12 (76)
Food awaiting sale is covered	3 (20)
Utensils used by the vendor are clean	12 (67)
Utensils used by the vendor are covered	6 (40)

**Table 3:** Microbial counts (log_10_ CFU/g) of street-vended chicken and food contact surfaces and equipment. Each data point represents mean ± SD of triplicates (*n* = 15).

Portion	*Salmonella*	*Escherichia coli*	*Campylobacter jejuni*	*Staphylococcus aureus*
Raw chicken	6.42 ± 1.64^a^	6.60 ± 1.25^a^	8.95 ± 0.94^a^	6.92 ± 1.32^a^
Cooked chicken	2.22 ± 1.88^b^	2.67 ± 1.98^b^	4.66 ± 2.67^d^	2.86 ± 1.61^c^
Hand	3.53 ± 2.17^b^	3.74 ± 1.92^b^	6.48 ± 0.99^b^	4.85 ± 1.00^b^
Knife	2.26 ± 1.63^b^	2.42 ± 1.48^b^	5.36 ± 0.43^cd^	4.00 ± 0.55^b^
Food contact surface	3.68 ± 1.82^b^	3.10 ± 1.92^b^	6.84 ± 0.71^b^	4.83 ± 0.88^b^
Storage container	3.37 ± 1.75^b^	3.77 ± 1.54^b^	6.11 ± 1.04^bc^	4.24 ± 0.95^b^

Values with different superscripts along a column are statistically different at *p* < 0.05.

## Data Availability

Confidentiality was assured to the participants but the data can be provided in case of request however after removal of any information that may breach the confidentiality of the participants.
